# Impact of overweight and obesity on pregnancy outcomes in women with gestational diabetes – results from a retrospective multicenter study

**DOI:** 10.20945/2359-3997000000178

**Published:** 2019-09-25

**Authors:** Catarina Machado, Sara Monteiro, Maria João Oliveira, Grupo de Estudo de Diabetes e Gravidez da Sociedade Portuguesa de Diabetologia

**Affiliations:** 1 Departamento de Endocrinologia Centro Hospitalar de Vila Nova de Gaia Espinho Porto Portugal Departamento de Endocrinologia, Centro Hospitalar de Vila Nova de Gaia/Espinho, Porto, Portugal

**Keywords:** Gestational diabetes, obesity, overweight, pregnancy outcome

## Abstract

**Objective:**

The aim of this study was to evaluate the impact of pre-pregnancy body mass index (BMI) on pregnancy outcomes in women with gestational diabetes (GD).

**Subjects and methods:**

Retrospective multicenter study using data from the Portuguese National Register. We included women with GD with a singleton pregnancy. GD diagnosis was according to the International Association of the Diabetes and Pregnancy Study Group criteria. Women were divided into groups according to their pre-pregnancy BMI: < 18.5 kg/m^2^ (underweight), ≥ 18.5 and < 25.0 kg/m^2^ (normal weight), ≥ 25 and < 30 kg/m^2^ (overweight) and ≥ 30 kg/m^2^ (obese).

**Results:**

We included 3,103 pregnant women with GD, 29.6% (n = 918) were overweight and 27.3% (n = 846) were obese. Compared to normal weight, the overweight and obese groups had a higher percentage of gestational hypertension (4.0% and 8.5% vs. 2.1%), cesarean delivery (32.8% and 41.3% vs. 27.9%), macrosomia (3.9% and 6.7% vs. 2.4%), and large for gestational age (LGA) newborns (8.3% and 13.5% vs. 6.0%). Obesity increased the risk of gestational hypertension (OR 4.5, p < 0.001), preeclampsia (OR 1.9, p = 0.034), cesarean delivery (OR 2.0, p < 0.001), macrosomia (OR 3.1, p < 0.001) and LGA (OR 2.3, p < 0.001).

**Conclusion:**

In pregnant women with GD, pregnancy complications increase with pre-pregnancy BMI. In obese women, appropriate diet and counseling prior to gestation and more aggressive medical intervention during pregnancy are needed in order to prevent macrosomic and LGA newborns and to reduce maternal complications.

## INTRODUCTION

Traditionally, gestational diabetes (GD) is a term used to describe the presence of glucose intolerance with onset or first detected during pregnancy (1). More recently, the American Diabetes Association defined GD as diabetes diagnosed in the second or third trimester of pregnancy in a patient without prior history of overt diabetes (2). Regardless of the definition of GD or the chosen screening criteria, it is the most common metabolic pregnancy complication (3), and its incidence is increasing, as the epidemic of obesity and type 2 diabetes mellitus (DM^2^) continues worldwide (2,4-6). There have been studies reporting that half of women are overweight or obese at the beginning of their pregnancies (6-10).

Maternal hyperglycemia in GD is associated with adverse maternal, fetal, and neonatal outcomes, when compared with women with no history of GD (4,11-13). Importantly, maternal obesity is independently associated with adverse maternal and neonatal outcomes (9,11,14), and increasing BMI is a well-known risk factor for the development of GD (6). There is an increased risk of preeclampsia and caesarean section for the mother, and an increased risk of macrosomia, LGA, congenital birth defects, or postnatal hypoglycemia for the fetus (4,11-13), among others. Moreover, the combination of GD and obesity is associated with more adverse pregnancy outcomes compared to GD or obesity alone (10). Recently, a study showed that women with GD and obesity had an increased risk of cesarean section, preeclampsia, and maternal morbidity when compared to either non-obese patients with GD and obese patients without GD (15).

As the prevalence of GD and obesity is increasing worldwide, the aim of this study was to evaluate the impact of pre-gestational BMI on pregnancy outcomes in women with GD.

## SUBJECTS AND METHODS

We retrospectively reviewed the Portuguese National Register of women with GD between January 1, 2016, and December 31, 2016. This database included women with no prior diagnosis of diabetes mellitus who received prenatal care in 26 medical institutions of the National Health System (NHS) in Portugal.

The diagnosis of GD was established following the recommendations from the Portuguese Society of Diabetologia (16). First, fasting blood glucose levels (FBG) were evaluated during the first trimester in all women; results of ≥ 92 mg/dL were diagnostic of GD, and an oral glucose tolerance test (OGTT) was not performed in these women. A 75-g 2-hour OGTT was performed in all women with normal first trimester FBG, between 24-28 weeks of gestation, with OGTT thresholds according to the International Association for Diabetes in Pregnancy Study Group’s (IADPSG) (17): fasting glucose ≥ 92mg/dL, 1-hour ≥ 180mg/dL and 2-hour ≥ 153 mg/dL. FBG and OGTT were not repeated, according to the recommendations from the Portuguese Society of Diabetologia (16). Women with FBG ≥ 126 mg/dL or random/2-hour following OGTT glucose levels ≥ 200 mg/dL were diagnosed with overt diabetes mellitus and were excluded from this study. We only included women with a singleton pregnancy. Women with missing information regarding initial BMI, FBG, or OGTT values and pregnancy outcomes were excluded from this study.

Women with GD were divided into four groups according to their pre-pregnancy BMI: < 18.5 kg/m^2^ (underweight), ≥ 18.5 and < 25.0 kg/m^2^ (normal weight), ≥ 25 and < 30 kg/m^2^ (overweight) and ≥ 30 kg/m^2^ (obese). The weight gained during pregnancy was evaluated based on pre-pregnancy BMI according to the Institute of Medicine (IOM) recommendations: range of adequate weight gain in pregnancy in underweight women between 12.5 and 18.0 kg, in normal weight women between 11.5 and 16.0 kg, in overweight women between 7.0 and 11.5 kg, and in obese women between 5.0 and 9.0 kg (18).

Pregnancy outcomes were defined according to IADPSG (19). We evaluated preeclampsia, gestational hypertension, hydramnios, preterm delivery, induction of labor, caesarean section, gestational age at delivery, fetal birth weight, fetal macrosomia, LGA, small for gestation age (SGA), neonatal hypoglycemia, neonatal hyperbilirubinemia, fetal congenital birth defects, admission in an intensive care unit, and fetal death.

The statistical analysis was performed with SPSSv20^®^. The Kolmogorov-Smirnov test of normality was used to analyze all data for normality of distribution. Continuous variables were expressed as the mean ± SD if normally distributed, or as median and interquartile range (IQR) if not normally distributed. Categorical variables were displayed as frequencies using the Fisher’s exact test or the chi-square test. For continuous variables, the appropriate parametric (Student t-test) or non-parametric (Mann-Whitney) test was used. Logistic regressions were performed to access the odds ratios (OR) for the maternal and fetal adverse outcomes using normal weight (18.5 ≤ BMI < 25.0) as the reference group. The results of logistic regression are presented as adjusted OR with the 95% confidence interval (CI), which included maternal age, education, and parity as covariates. Statistical significance was awarded if p value < 0.05.

## RESULTS

**Figure 1:**
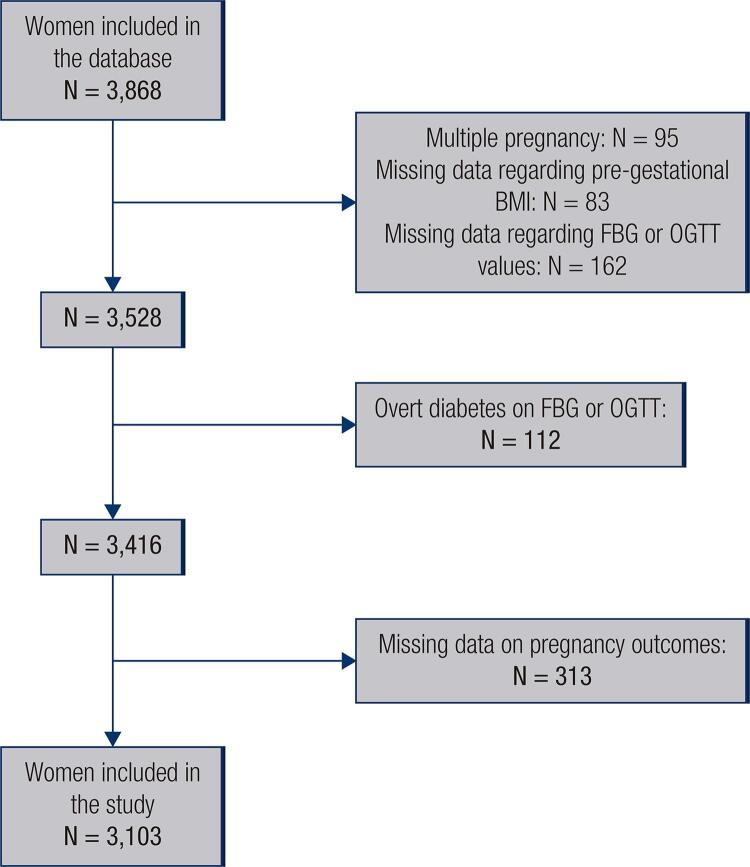
Flowchart of patient inclusion and exclusion in the study.

A total of 3,868 women were registered on our database. Of these, 3,103 met inclusion criteria and were included in this study (Figure 1). Women were divided into four categories according to their pre-pregnancy BMI: 29.6% women (n = 918) were classified as overweight and 27.3% (n = 846) as obese. Mean age of the overweight patients was higher compared to normal weight and obese women. A total of 1,217 women (39.2%) required pharmacological treatments (30.2% insulin, 5.4% metformin and 3.5% insulin plus metformin), and 1,886 (60.8%) continued on diet and lifestyle modifications throughout pregnancy. Most women (n = 1729, 55.7%) were diagnosed following an abnormal OGTT. Maternal characteristics in relation to BMI are shown in Table 1.

**Table 1:** Maternal characteristics in relation to BMI

	BMI			

	Underweight n = 63 (2.0%)	Normal weight n = 1,276 (41.1%)	Overweight n = 918 (29.6%)	Obese n = 846 (27.3%)
Mean age (years)	30.4 ± 6.0*	33.2 ± 5.5	33.6 ± 5.3*	33.3 ± 5.4
Maternal education – n (%)				
No education	0 (0.0)	9 (0.8)	9 (1.2)	2 (0.3)^†^
Primary education	28 (52.8)*	297 (27.1)	268 (34.5)*	312 (42.2)*^†^
Secondary education	10 (18.9)*	358 (32.6)	254 (32.7)	262 (35.5)
Post-secondary education	15 (28.3)	433 (39.5)	246 (31.7)*	163 (22.1)*^†^
Nulliparity – n (%)	40 (63.5)*	631 (49.5)	359 (39.2)*	291 (34.4)*^†^
Previous DG – n (%)	3 (11.5)	136 (18.2)	121 (19.3)	123 (20.5)
Previous macrosomia – n (%)	1 (3.8)	41 (5.5)	62 (9.9)*	54 (9.0)*
Family history of diabetes – n (%)	25 (41.0)	507 (40.5)	428 (47.4)*	422 (51.8)*^†^
Median weight gained (kg)	13.0 [9.0 – 16.3]	11.3 [8.9 – 15.0]	10.0 [6.0 – 13.5]*	7.0 [3.0 – 11.1]*^†^
Excessive weight gained – n (%)	11 (19.0)	206 (17.1)	319 (36.7)*	290 (36.9)*
Inadequate weight gained – n (%)	28 (48.3)	606 (50.2)	239 (27.5)*	266 (33.8)*
Diagnosis – n (%)				
Fasting plasma glucose levels 1^st^ trimester	26 (41.3)	519 (40.7)	392 (42.7)	437 (51.7)*^†^
OGTT	37 (58.7)	757 (59.3)	526 (57.3)	409 (48.3)*^†^
Median gestational age at diagnosis (week)	24 [9 – 26]	24 [9 – 26]	24 [9 – 26]	18 [8 – 25]*†
Median fasting glucose levels 1^st^ trimester (mg/dL)	94.0 [92.0 – 99.3]	94.0 [93.0 – 97.0]	95.0 [93.0 – 99.0]*	96.0 [93-0 – 100.0]*^†^
Median glucose levels on 75-g OGTT (mg/dL)				
0’	75.0 [69.8 – 79.8]	79.0 [73.0 – 88.0]	83.0 [76.0 – 92.0]*	87.0 [79.0 – 94.0]*^†^
60’	181.0 [158.0 – 189.0]	180.0 [157.0 – 190.0]	181.0 [156.0 – 193.0]	181.0 [162.0 – 192.0]*
120’	156.0 [143.5 – 162.5]	156.0 [131.0 – 165.0]	155.0 [131.0 – 169.0]	151.0 [124.0 – 166.0]*^†^
Diet and lifestyle modification – n (%)	53 (84.1)*	912 (71.5)	530 (57.7)*	391 (46.2)*^†^
Pharmacological treatment – n (%)	10 (15.9)*	364 (28.5)	388 (42.3)*	455 (53.8)*^†^
Insulin	10 (90.0)	292 (80.2)	294 (75.8)	343 (75.4)
Metformin	0 (0.0)	54 (14.8)	59 (15.2)	56 (12.3)
Insulin plus metformin	1 (10.0)	18 (4.9)	35 (9.0)	56 (12.3)

* *p* < 0.05 *vs*. normal weigh (BMI < 18.5–24.9); ^†^*p* < 0.05 *vs*. overweight (BMI 25.0–29.9).

Pregnancy outcomes in GD according to pre-pregnancy BMI are shown in Table 2. The prevalence of preeclampsia and gestational hypertension was higher in the obese group (3.9% and 8.5%, respectively). The median gestational age at delivery was similar between all groups [39 weeks (IQR 38–40 in the underweight group and IQR 38-39 in the normal weight, overweight, and obesity groups)], even though preterm delivery was more prevalent in the overweight group, compared to the normal weight and obese groups. Overweight and obese women had less spontaneous delivery, and the rate of caesarean section was highest in the obese women (41.3%), followed by the overweight (32.8%). Fetal birth weight was higher in the overweight and obese groups, so was the prevalence of LGA. The incidence of SGA was highest in the underweight group (14.3%). Congenital birth defects, hypoglycemia, hyperbilirubinemia, fetal death, or need for admission in an intensive care unit were similar between groups.

**Table 2:** Pregnancy outcomes in GD according to pre-pregnancy BMI

	BMI			

	Underweight n = 63 (2.0%)	Normal weight n = 1,276 (41.1%)	Overweight n = 918 (29.6%)	Obese n=846 (27.3%)

Maternal complications				
Gestational hypertension – n (%)	1 (1.6)	26 (2.1)	35 (4.0)*	65 (8.5)*^†^
Preeclampsia – n (%)	1 (1.6)	25 (2.0)	26 (2.8)	33 (3.9)*
Hydramnios – n (%)	2 (3.2)	22 (1.7)	17 (1.9)	22 (2.6)
Preterm delivery – n (%)	6 (9.5)	85 (6.7)	80 (8.7)*	48 (5.7)^†^
Induction of labor – n (%)	26 (41.3)	446 (35.0)	366 (39.9)*	391 (46.2)*^†^
Caesarean delivery – n (%)	15 (23.8)	356 (27.9)	301 (32.8)*	349 (41.3)*^†^

**Fetal and neonatal complications**				

Median birth weight (grams)	3040.0 [2680.0 – 3285.0]	3120.0 [2820.0 – 3410.0]	3240.0 [2945.0 – 3511.0]*	3285.0 [2980.0 – 3590.0]*^†^
Macrosomia – n (%)	0 (0.0)	30 (2.4)	36 (3.9)*	57 (6.7)*†
LGA – n (%)	3 (4.8)	77 (6.0)	76 (8.3)*	115 (13.6)*^†^
SGA – n (%)	9 (14.3)	124 (9.7)	65 (7.1)*	61 (7.2)*
Neonatal hypoglycemia – n (%)	2 (3.2)	58 (4.5)	46 (5.0)	34 (4.0)
Neonatal hyperbilirubinemia – n (%)	8 (12.7)	134 (10.5)	109 (11.9)	104 (12.3)
Congenital defects – n (%)	1 (1.6)	45 (3.5)	33 (3.6)	27 (3.2)
Admission in an intensive care unit – n (%)	5 (7.9)	74 (5.8)	60 (6.5)	61 (7.2)
Fetal death – n (%)	0 (0.0)	1 (0.1)	1 (0.1)	1 (0.1)

* *p* < 0.05 *vs*. normal weigh (BMI <18.5–24.9); ^†^*p*<0.05 *vs*. overweight (BMI 25.0–29.9).

In Table 3, the pregnancy outcomes are shown according to the pre-pregnancy BMI. Obese women had an increased risk of gestational hypertension, preeclampsia, and cesarean delivery (OR: 4.5, 1.9, and 2.0, respectively). A spontaneous delivery was less likely to occur in overweight and obese patients. A higher pre-pregnancy BMI increased the risk of macrosomia and LGA newborns.

**Table 3:** Logistic regression of pregnancy outcomes by maternal BMI

Outcome	Underweight OR [IC 95%]	p-value	Normal weight OR [IC 95%]	Overweight OR [IC 95%]	p-value	Obese OR [IC 95%]	p-value
Gestational hypertension	1.118 [0.143; 8.764]	0.916	1.0	1.851 [1.061; 3.229]	0.030*	4.473 [2.681; 7.462]	0.001*
Preeclampsia	--	--	1.0	1.321 [0.734; 2.392]	0.351	1.872 [1.049; 3.343]	0.034*
Hydramnios	0.487 [0.104; 2.291]	0.362	1.0	0.915 [0.456; 1.837]	0.804	1.323 [0.696; 2.516]	0.393
Preterm delivery	0.553 [0.207; 1.478]	0.237	1.0	1.702 [1.201; 2.410]	0.003*	0.908 [0.602; 1.370]	0.646
Cesarean delivery	1.269 [0.634; 2.539]	0.501	1.0	1.268 [1.035; 1.553]	0.022*	1.963 [1.598; 2.410]	0.001*
Induction of labor	0.820 [0.462; 1.458]	0.499	1.0	1.235 [1.021; 1.495]	0.030*	1.635 [1.345; 1.988]	0.001*
Macrosomia	--	--	1.0	1.831 [1.048; 3.200]	0.034*	3.140 [1.873; 5.265]	0.001*
LGA	0.622 [0.380; 6.920]	0.513	1.0	1.431 [1.001; 2.044]	0.049*	2.260 [1.613; 3.166]	0.001*
SGA	0.520 [0.232; 1.170]	0.114	1.0	0.635 [0.448; 0.901]	0.11*	0.689 [0.483; 0.983]	0.040*

*p < 0.05.

In addition to different pregnancy outcomes, the management of GD was different according to maternal BMI. Pharmacological treatment was progressively more needed as BMI increased (42.3% in the overweight group, p < 0.001, and 53.8% in the obese group, p < 0.001, compared to 28.5% in the normal weight group), and treatment with insulin was more frequent in obese (47.2%) and overweight (35.8%) women, compared to 24.3% in the normal weight group.

## DISCUSSION

In this study, more than half of the women (56.9%) who were diagnosed with GD were overweight or obese. This worrying percentage reflects the dramatic increase in overweight and obesity among women of reproductive age. There are some reports in the literature that showed similar results (3,9,20,21) with Ovesen and cols. and Scifres and cols. reporting a percentage of obese women with GD of more than 70.0% (22,23). Obesity and GD can lead to adverse outcomes, and, in our study, women who were overweight and obese prior to gestation had an increased risk of adverse pregnancy outcomes. Similar to reports in the literature, in our study, women who were overweight or obese were more often diagnosed with gestational hypertension or preeclampsia (11,15,20,21,23,24), labor was more likely to be induced (20), and the rate of cesarean section was higher (9,15,20,24,25).

The risk of developing gestational hypertension was significantly higher in overweight and obese women. In fact, the risk of gestational hypertension increased with increasing BMI, and obese women had a 4.5-fold risk of developing gestational hypertension, as shown in Table 3. We reported a prevalence of 8.5% gestational hypertension in the obese group, which was higher when compared to the normal weight and overweight groups. Some studies similar to ours showed higher rates of pregnancy-induced hypertension in the obese women: Sugiyama and cols. reported a 14.8% rate of pregnancy-induced hypertension (20), Miao and cols. 11.4% (24), and Sun and cols. 11.8% (11). Pregnancy-induced hypertension complicates 6–10% of all pregnancies (26), and GD is a well-known risk factor for the development of hypertension (13). Apart from GD, other factors, such as maternal age or ethnicity, could explain the development of hypertension (27), however, the association with pre-pregnancy BMI is well established (3,28).

The overall rate of cesarean delivery in our study was 32.9%, a higher percentage compared to the rate of cesarean sections in the Portuguese NHS hospitals in 2016 (23.5%) (29). Compared to the normal weight group, there was a higher rate of cesarean delivery in the overweight and obese groups (32.8% and 41.3%, respectively), possibly in relation to increased macrosomic and LGA newborns. Both obesity and GD are independent risk factors for cesarean delivery (6,12,15). In this study, there was a progressive increase in OR with the increasing BMI category regarding cesarean section. In some studies, the cesarean delivery rate reported in obese women with GD was approximately 40.0% (6,15,20), with one study reporting a 72.7% rate with a 3.2-fold increased risk of cesarean section (OR 3.26; IC 95% 1.57–6.76, p = 0.002) (24). The high percentage of cesarean delivery in this population could be due to suspected macrosomia or failure to induce labor.

Regarding neonatal outcomes, similar to what was described in previous reports, newborns of overweight and obese women were more likely to be macrosomic (11,21,23-25) and LGA (20,21,23,24). This is of particular importance once these infants have higher risk of future development of metabolic syndrome and cardiovascular disease (13,20,30,31). One of the reasons that could explain this is an inadequate glycemic control in the overweight and obese women, thereby exposing the fetus to hyperglycemia. Diet modification is the primary choice of treatment in GD, with pharmacological approaches used when it fails (11). Because pharmacologic treatment was more often needed in these groups, the assumption they had worse glycemic control can be speculated. However, data on glycemic control was not available, therefore, we cannot establish whether glycemic control was achieved in any of the groups. Another explanation could be gestational weight gain. Excessive weight gained is associated with an increased risk of fetal overgrowth in obese women (23), and, in this study, 36.9% of obese women gained excessive weight according to the IOM recommendations. Although there was an increased risk of LGA infants born from obese women, the frequency of this adverse fetal outcome in our study (13.6%) was lower compared to similar studies that reported a percentage of approximately 20.0% (6,15).

In our study, GD was diagnosed earlier in overweight and obese women. More specifically, the obese group was more often diagnosed following an elevated fasting blood glucose level during the first trimester of pregnancy, whilst the other three groups were diagnosed later in pregnancy following an abnormal OGTT. The median fasting glucose levels were superior in overweight and obese women, but, contrasting with other reports, glucose levels following OGTT were similar between the four groups. Previous reports in the literature showed a gradual increase in glucose levels on 75g OGTT according to pre-pregnancy BMI (20,32). This is possibly a result of different diagnostic methods, as in most studies an OGTT after the 24^th^ week of gestation is the only screening approach. In our study, an elevated fasting blood glucose level at the first trimester made the diagnosis of GD in 42.7% of the overweight women and in 51.7% of the obese women. For that reason, the OGTT was not performed in a considerable proportion of women with overweight and obesity. This difference in screening methods could also explain the differences between our study and similar reports, regarding the lower frequencies of gestational hypertension (11,15), preeclampsia (6), macrosomia (6,11,23), and LGA newborns (6,15,20). Impaired fasting glucose levels due do insulin resistance and increased liver gluconeogenesis are common in obese women and may be present early in gestation. As obese women were more often diagnosed in the first trimester of pregnancy, this may have led to a prompt approach in order to restore euglycemia, thereby reducing the incidence of these adverse outcomes. FBG as a screening test for GD in the first trimester of pregnancy is not widely accepted, as many expert panels advocate the 2-hour OGTT at 24–28 weeks of gestation for all pregnant women. As a diagnostic tool, FBG presents some limitations as it seems to be more sensitive than specific for the screening of GD (33,34). Physiologically, there is a fall in FBG in early pregnancy, so mild hyperglycemia at the beginning of the pregnancy may represent early glucose intolerance. As there is a continuous relation between maternal hyperglycemia and adverse pregnancy outcomes (12), we believe FBG should be evaluated in all pregnant women during the first trimester, especially if overweight or obese, in order for early detection of GD and to prevent possible adverse outcomes.

Gestational weight gain in the obese and overweight group was inferior compared to normal weight and underweight women, which is concordant with other studies (20,21,23). This suggests a recognition of the potential risks overweight and obese women face during pregnancy and, possibly, a more intensive intervention in these women. However, regarding their initial BMI, a considerable percentage of women in the overweight and obese groups still had an excessive weight gain, suggesting a need to improve counseling prior to conception, in order to reduce the percentage of women with excessive body weight at the beginning of the pregnancy. The gestational weight gained during pregnancy was different between women diagnosed in the first trimester of pregnancy and women diagnosed following the OGTT [9.2kg (IQR 6-13) vs. 10.5 kg (IQR 7-14), p < 0.001). An early implementation of diet and lifestyle modification following the diagnosis of GD may have ameliorated excessive weight gain in women diagnosed in the first trimester.

Our study has several strengths and limitations. A major strength is it included a large multicenter cohort of women with GD. We also show evidence for the clinical relevance of screening for GD in the first trimester of pregnancy, supporting the IADPSG recommendations regarding early identification of GD.

The main limitation of this study lies within its retrospective design. Retrospective studies are subject to the quality of information available, and information may be incomplete. In this case, our database lacked information on maternal ethnicity, household income, socioeconomic class, or smoking status. These maternal characteristics could influence pregnancy outcomes. Another limitation is the lack of data on the adequacy of glycemic control and on dietary plans. In our study, we couldn’t access glycemic control or dietary plans among pregnant women with GD with and without overweight/obesity. Uncontrolled hyperglycemia is a well-known risk factor for maternal and fetal adverse outcomes, and Huett and cols. reported an increased rate of induced labor, macrosomia, and LGA newborns in uncontrolled GD that was independent of obesity (15).

This study only included women with GD. As the epidemic of obesity rises, it is important to compare pregnancy outcomes in overweight and obese women with and without GD, in order to access the influence of both maternal hyperglycemia and obesity. For instance, Martin and cols. reported an increased risk of cesarean delivery and LGA newborns in obese women that was independent of GD (6); Blickstein and cols. suggested the risk of preeclampsia and macrosomia was influenced by obesity only (25); and, more recently, Huett and cols. described a higher risk of macrosomia in obese women without GD compared to obese women with GD and a significantly increased risk for cesarean section and maternal morbidity in obese women, regardless of the control of diabetes (15).

In conclusion, this study provides evidence maternal pre-pregnancy BMI influences pregnancy outcomes in women with GD, as previously shown in other reports. In our study of a large population of women with GD, pre-pregnancy BMI was an important determinant of adverse pregnancy outcomes, such as gestational hypertension, preeclampsia, induced labor, cesarean section, macrosomia, and LGA. Efforts to reduce overweight and obesity prior to conception and to reduce excessive weight gain in women with GD should be made, in order to avoid these adverse outcomes.
